# Reproductive Biology of the Two Varieties of *Anacyclus pyrethrum* L.—*Anacyclus* *pyrethrum* var. *pyrethrum* (L.) Link and *Anacyclus pyrethrum* var. *depressus* (Ball.) Maire—An Endemic Endangered Species

**DOI:** 10.3390/plants11172299

**Published:** 2022-09-01

**Authors:** Fatima Zahra Jawhari, Hamada Imtara, Abdelfattah El Moussaoui, Hind Khalis, Imane Es-Safi, Omkulthom Al Kamaly, Asmaa Saleh, Mohammad Khalid Parvez, Raja Guemmouh, Amina Bari

**Affiliations:** 1 Laboratory of Biotechnology, Environment, Agri-Food and Health (LBEAS), Faculty of Sciences, University Sidi Mohamed Ben Abdellah (USMBA), B.P. 1796, Fez 30003, Morocco; abdelfattah.elmoussaoui@usmba.ac.ma (A.E.M.); imane.safi@usmba.ac.ma (I.E.-S.); amina.bari@usmba.ac.ma (A.B.); 2 Faculty of Arts and Sciences, Arab American University Palestine, Jenin P.O. Box 240, Palestine; 3 Laboratory of Geodynamics and Natural Resources LGRN, Faculty of Sciences, University of Sidi Mohamed Ben Abdullah, (USMBA), B.P. 1796, Fez 30003, Morocco; hind.khalis@usmba.ac.ma; 4 Department of Pharmaceutical Sciences, College of Pharmacy, Princess Nourah bint Abdulrahman University, P.O. Box 84428, Riyadh 11671, Saudi Arabia; omalkmali@pnu.edu.sa (O.A.K.); asmaa.saleh@usmba.ac.ma (A.S.); 5 Department of Pharmacognosy, College of Pharmacy, King Saud University, P.O. Box 2457, Riyadh 11451, Saudi Arabia; mohammad.parvez@usmba.ac.ma; 6 Laboratory of Biotechnology, Conservation and Valorisation of Natural Resources (LBCVR), Faculty of Sciences, University Sidi Mohamed Ben Abdellah (USMBA), Fez 30003, Morocco; raja.guemmouh@usmba.ac.ma

**Keywords:** breeding system, pollinators, phenology of flowering, seed dispersal

## Abstract

The reproductive system is essential for the structuring and transmission of genetic diversity. Understanding the reproductive biology of threatened endemic species is considered to be a crucial element for the implementation of effective conservation strategies. Given the lack of information and the insufficient state of knowledge on the reproductive system of *Anacyclus pyrethrum* L., a threatened medicinal species endemic to Morocco, we are the first to study the reproductive biology of two varieties of *Anacyclus pyrethrum* L.: *Anacyclus pyrethrum* var. *pyrethrum* (L.) Link and *Anacyclus pyrethrum* var. *depressus* (Ball.) Maire. The reproductive biology of the two varieties was examined in detail by studying the development of the inflorescence, phenology of flowering, breeding system, pollinators, production, and seed dispersal. The experimental results described in this work suggest that *Anacyclus pyrethrum* L. is a gynomonic species, with a mixed autogamy–allogamy reproductive regime with a high predominance of allogamy. It appears to be partially self-incompatible, with allogamy rates for *Anacyclus pyrethrum* var. *depressus* (Ball.) Maire and *Anacyclus pyrethrum* var. *pyrethrum* (L.) Link of 78.70% and 79.01%, respectively. It depends on pollination vectors to produce a large number of seeds. This study on the breeding system of *Anacyclus pyrethrum* L. provides a tool for developing management strategies and adequate conservation measures.

## 1. Introduction

Reproductive biology is an important and critical trait as it characterises growth and development, and plays a role in the implementation of conservation strategies [1,2]. Plant reproduction involves either a sexual mode, resulting in variable seeds reflecting the genetic contribution of both parents; an asexual mode, including vegetative propagation and/or clonal seed formation (apomixis), in which the genetic characteristics of the parent plant are retained; or a combination of both [3]. Reproductive biology is the study of floral phenology and the reproductive system (sexual systems, apomixis, incompatibility mechanisms, reproduction regime, pollination, and germination) [4,5]. The reproductive system plays a crucial role in structuring and transmitting genetic diversity as well as in determining the levels of loss of this diversity [6]. Understanding the reproductive biology of endemic and threatened species is crucial to the development of effective conservation programmes [7,8].

For a long time, the diversity of reproductive systems observed in plants has fascinated biologists [9,10,11]. The variability in the reproductive systems found in angiosperms is the result of the coexistence of different sexual phenotypes, with systems of one sexual phenotype and systems with two or more sexual phenotypes. In angiosperms, the majority of species are hermaphroditic (72%) (both female and male sexes are present in the same flower) and the minority (4%) are dioecious (male and female individuals). Between these two systems there are androdioecy (the presence of male and hermaphroditic individuals in the same population), gynodioecy (the presence of female and hermaphroditic individuals in the same population), andromonoecy (male and hermaphroditic flowers in the same individual), and gynomonoecy (female and hermaphroditic flowers in the same individual) [10,11]. The proportions of different sexual phenotypes may vary between populations within a species [10]. Within the Asteraceae family, gynomonoecia is the reproductive system most frequently represented [12,13].

Historically, the identification of Asteraceae has been based on their capitula, with flowers closely arranged on a receptacle surrounded by bracts [14,15,16,17]. Structurally, the capitula are synchronised to function as an efficient reproductive unit [17,18]. The frequent arrangement of the capitulum of the Asteraceae is formed by actinomorphic disc flowers that assume a reproductive function, thus improving the chances of reproduction and facilitating a more flexible basis for the evolution of the reproductive system as well as peripheral zygomorphic flowers that are highly specialised in attracting pollinators [16,17]. The variation in the expression of floral sexuality associated with changes in symmetry has important consequences for the evolution of reproductive biology in the family [12,17,19].

*Anacyclus pyrethrum* L. is a medicinal species endemic to Morocco, Algeria, and Spain belonging to the Asteraceae family genus Anacyclus. In 1979, the species was identified with two varieties: *Anacyclus pyrethrum* var. *pyrethrum* (L.) Link (*A.P* var. *pyrethrum*) and *Anacyclus pyrethrum* var. *depressus* (Ball.) Maire (*A.P* var. *depressus*) [20,21,22]. The roots are the most used part of the plant, which is known for its various therapeutic traits as a sialagogue [23,24,25], an aphrodisiac [26,27,28,29,30,31], n immunostimulant [32,33], an antidepressant [34], and a muscle relaxant [35] as well as its antimicrobial [36,37,38,39], antibacterial [40,41], antifungal [42], insecticide [43,44,45], local anaesthetic [46], anti-inflammatory [47,48,49], anticonvulsant [26,50,51,52], antioxidant [31,41,51,53,54], antidiabetic [40,55,56,57], androgenic and fertiliser [30,58], antiamnesic [51], and anticancer [59] properties. It is also used as a memory stimulant [60].

The excessive overexploitation of the roots of the plant for trade makes it very local, uncommon, and quite rare in most of its known sites. Recently, it has been placed in the ‘vulnerable’ category according to the IUCN red list and the red list of vascular plants in Morocco. 

Given the insufficient state of knowledge and the lack of information on the reproductive biology of the two varieties of *Anacyclus pyrethrum* L., the objective of this study was to analyse its reproductive biology; specifically, to examine the development of the inflorescence, the phenology of flowering, the reproductive system, and the production and dispersal of seeds as well as the insect pollinators. The ultimate aim was to understand the implications of these data in evaluating future breeding and conservation strategies for the studied species. The questions we attempted to answer were:

− What is the reproductive system of *Anacyclus pyrethrum* L.?
− Are pollinating insects necessary for the two varieties to be successful in producing seeds?

## 2. Results and Discussion

### 2.1. Population Density

After setting up quadrats, the number of individuals within the limits of each quadrat was counted. The size of each population is represented in Table 1. Several quadrat samples were taken at random locations, which ensured that the recorded numbers were representative of the study station as a whole.

The results showed that the individuals constituting each population were randomly dispersed according to an aggregate dispersal pattern, and that the density of plants in 2019 was significantly lower than observed in 2018, with the exception of the Ifrane station where we observed an increase in the number of individuals. This increase was explained by the protection of this population due to its proximity to the royal palace. This confirmed that the number of individuals increased in legally protected populations.

A comparison of the different quadrats revealed that the plant density was significantly higher in the quadrats closest to habitations, indicating that individuals closer to habitations were more protected. The plant density per quadrat was also significantly different between the two varieties, with *A.P* var. *depressus* being more frequent than *A.P* var. *pyrethrum*. The plant density in 2019 was low compared with 2018; these differences were mainly due to the collection process. In recent years, local collectors have reported a significant decline in both varieties of *Anacyclus pyrethrum* L., aggravated by premature harvesting (before the seed dispersal season); *A.P* var. *pyrethrum* is the variety most affected by this overexploitation. Our results on the decline of *A.P* var. *pyrethrum* in the Middle Atlas were consistent with those of Ouarghidi, who showed a significant decline in *A.P* var. *pyrethrum* populations in southern Morocco (Ait Ahmed valley) [21,22].

### 2.2. Phenology and Fecundity of Natural Populations

The natural populations remain dormant as underground rhizomes throughout the winter; they start to develop from the fourth week of March and continue until the end of the vegetative phase and the beginning of the sexual phase (inflorescence) in the third week of April. The formation of floral buds continues until the fourth week of June; the flowers of the same plant do not flower at the same time. At the capitulum level, the flowering is centripetal; the florets open from the periphery of the floral ray toward the centre and they remain fully open for about 4 weeks after the emergence of the floral bud. Senescence takes place during July and August; during these months, all aerial parts dry out and the dry flower heads are dispersed. Seeds do not immediately disintegrate from the capitula. Under greenhouse conditions, the activation of the vegetative phase starts in the first week of March and enters the sexual phase (appearance of the first flowers) at the latest from the fourth week of May until the first or second week of July. The beginning of flowering is signalled by the centripetal blooming of the peripheral ray florets and the continuation of the tubular florets. The plants begin to senesce by the latest at the end of July and continue until the second week of August.

**Figure 1:**
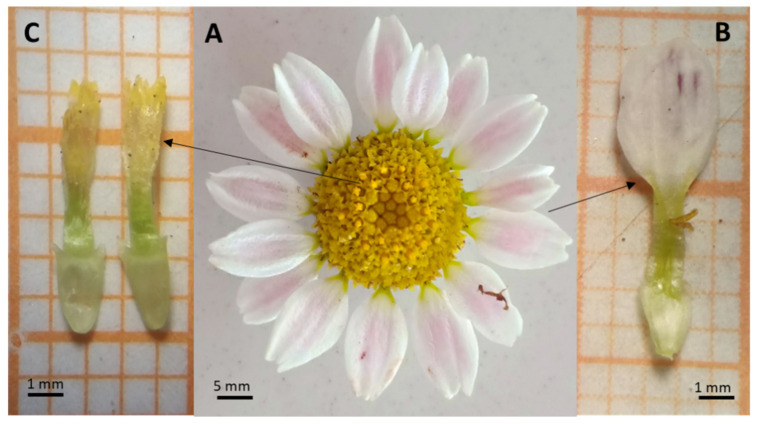
Floral organisation of the study species. (**A**) Flower head; (**B**) ligulate flower; (**C**) tubular flower.

The species is known by a slightly flattened solitary capitulum with a diameter varying between 7 mm and 12 mm for *A.P* var. *depressus* and from 13 mm to 23 mm for *A.P*. var. *pyrethrum* (Figure 1A). The involucre bracts, arranged in 3 series, are lanceolate, glabrous, scarieuses, and coloured green. The floral receptacle bears peripheral ray florets in a single row and are zygomorphic and female with tridentate corollas; they are white with a purple back in *A.P*. var. *depressus* and red in *A.P*. var. *pyrethrum*, averaging 9 mm in length and 2.4 mm in width in *A.P*. var. *depressus* and 15 mm in length and 3.19 mm in width in *A.P*. var. *pyrethrum* (Figure 1B). The capitulum centre is formed by several tubular flowers measuring about 7.2 mm in length in the variety *A.P*. var. *pyrethrum* and 4.2 mm in length in the variety *A.P*. var. *depressus*. They are actinomorphic, hermaphroditic, and yellow with a corolla consisting of 5 welded petals, 5 stamens, and a style ending in a bifid stigma (Figure 1C). For both types of flowers, the ovary is inferior, bicarpellate, uniovulate, and unilocular, measuring from 1mm to 4mm in length depending on the variety.

The results showed that the variety *A.P*. var. *depressus* has a high number of capitula and, therefore, produces more seeds compared with the variety *A.P*. var. *pyrethrum*, which has a high number of seeds per capitulum, but produces fewer capitula. However, the results also showed that both varieties have a high fruiting percentage (90%) (Table 2).

### 2.3. Reproduction Biology

Under the experimental conditions in a greenhouse, the bagged capitula failed to form seeds. The capitula of bagged individuals only produced seeds from the female peripheral flowers whereas the non-bagged individuals produced seeds not only from the central (hermaphroditic), but also from the peripheral (female) flowers (Table 3).

The fructification index was nil for both varieties for the bagged capitula lot (lot 1), and 0.16 and 0.15 for *A.P*. var. *depressus* and *A.P*. var. *pyrethrum*, respectively, for the lot of bagged individuals (lot 2). The fructification index was 0.75 for *A.P*. var. *depressus* and 0.74 for *A.P*. var. *pyrethrum* for the free pollination lot (lot 3). The allogamy rate was 100% for lot 1; for lot 2, the allogamy rates for *A.P*. var. *depressus* and *A.P*. var. *pyrethrum* were 78.70% and 79.01%, respectively. Therefore, *Anacyclus pyrethrum* L. had a mixed self-pollinated–allogamous reproductive regime with a strong predominance of allogamy. We could deduce from these results that the absence of seeds in the bagged capitula lot showed that there was a self-incompatibility between the female peripheral flowers and the hermaphrodite central flowers in the capitula. In the case of the bagged individuals, only the peripheral female flowers produced seeds, showing intra-individual self-compatibility between the peripheral female flowers and the central hermaphrodite flowers, and intra-individual self-incompatibility between the central hermaphrodite flowers of the same individual.

In Asteraceae, mutations probably related to organ identity genes may have occurred independently during angiosperm evolution, leading to gynomonoecy as the main sexual system in all species with radiate capitula [61,62,63]. In addition to organ identity genes, CYC-like genes, or those that control floral symmetry, are also involved in the expression of gynomonoecy related to radiate capitula [17,61,64]. However, gynomonoecia is also found to be occasional or rare within a species, and has been interpreted as being caused by genetic and environmental factors [61,63,65,66]. In particular, the genus Anacyclus represents a partially self-incompatible gynomonic reproductive system [20,61,67]. These data explain the results we obtained to determine the reproduction system of *Anacyclus pyrethrum* L. and show that the two varieties, *A.P*. var. *depressus* and *A.P*. var. *pyrethrum*, are able to reproduce in xenogamy with a fructification index of 0.75 and 0.74, respectively. In geitonogamy, only the peripheral flowers produce fruit; we observed a fructification index of 0.16 in *A.P*. var. *depressus* and 0.15 in *A.P*. var. *pyrethrum*. However, fruit production is significantly higher in allogamy than in autogamy. In light of these results, we can state that *Anacyclus pyrethrum* L. is a gynomonoecious species and partially self-incompatible, with geitonogamous peripheral flowers that have adapted to the absence of pollinators and xenogamous central flowers. These results were in alignment with those of Humphries, who found that four populations of *A.P*. var. *pyrethrum* and *A.P*. var. *depressus* were self-incompatible; all attempts to cross *A.P*. var. *pyrethrum* or *A.P*. var. *depressus* with annual taxa of the same genus ended in failure [67]. A few studies have shown that there is a positive correlation between the population size and the rate of allogamy in populations [68]. Self-pollinating plants can produce offspring and establish a new population from a single individual and thus have reproductive assurance. In contrast, xenogamous plants (cross-pollination) need another plant and vectors for pollination. As a result, self-pollinating plants have higher rates of propagation than cross-pollinating plants in closely related taxa. This could be the reason why self-pollinating taxa have been shown to be more widespread than xenogamous taxa in many closely related taxa [69,70]. However, xenogamous plants can increase genetic heterogeneity and are, therefore, favoured in heterogeneous and variable environments. As for the reproduction system, estimates of the rates of allogamy and autogamy are often required for plant breeding, conservation, and management [71]. Different works have reported that *Anacyclus pyrethrum* L. can reproduce asexually from root cuttings [20,21,22,36]. The combination of vegetative reproduction and xenogamy may contribute to the wide distribution of this plant, which explains that the rarity of this species is not due to the reproductive system, but to the overexploitation of the species during the flowering season, which influences the natural regeneration of the species.

### 2.4. Pollen

**Figure 2:**
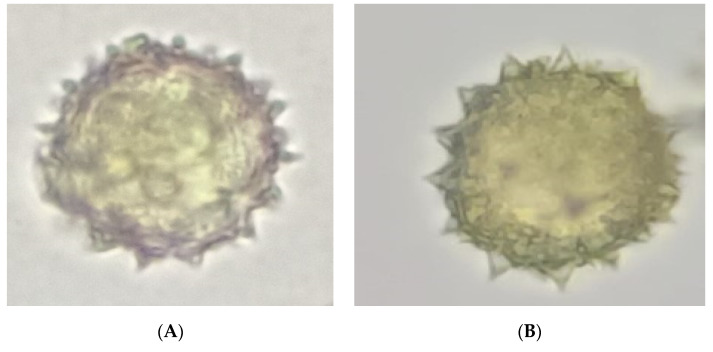
Pollen grain morphology of the two varieties *A.P*. var. *depressus* (**A**) and *A.P*. var. *pyrethrum* (**B**).

At anthesis, the stamens release a large quantity of pollen grains. Microscopic observations showed spheroidal pollen grains that were radially symmetrical, circular-polar, and equatorial in outline; they were echinulate with triangularly outlined spines tapering toward the tip and broad at the base, and densely arranged (Figure 2). This pollen type fits perfectly with the allogamous strategy and has adapted to be transported by pollinators.

### 2.5. Pollination in the Natural Environment

**Figure 3:**
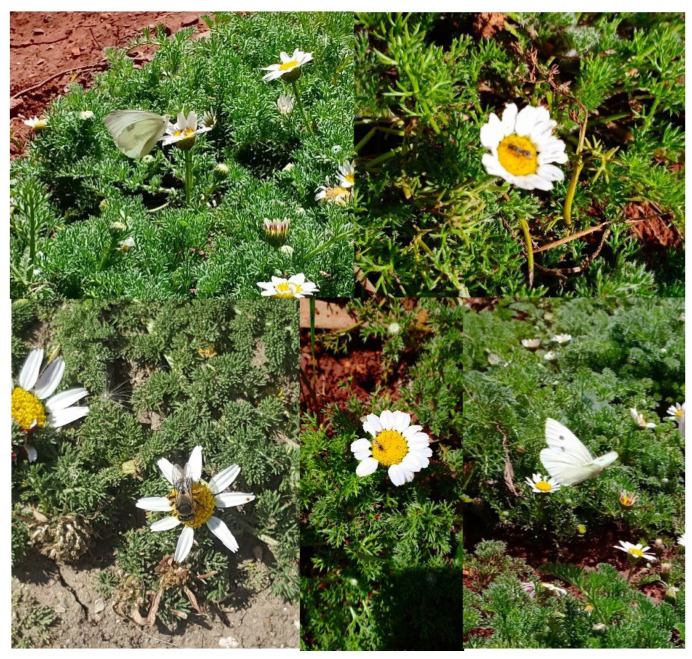
Examples of pollinators visiting the capitula of *A.P*. var. *pyrethrum* and *A.P*. var. *depressus*.

During the flowering period, several species of insects were observed on the capitula of *A.P*. var. *pyrethrum* and *A.P*. var. *depressus* (Figure 3).

The orders of the insects documented as pollinators in the present study belonged mainly to Hymenoptera, Coleoptera, and Orthoptera. Hymenoptera were the most frequent visitors to the study sites (Table 4).

Among the pollinators observed, species belonging to the orders Araneidae and Lepidoptera were identified only in the stations of the variety *A.P*. var. *pyrethrum*; Dermaptera were represented only in the stations of the variety *A.P*. var. *depressus*. Asteraceae are generally pollinated by several groups of insects such as Diptera, Coleoptera, and Hymenoptera [72,73,74,75,76] as the main function of ray flowers in most Asteraceae species is related to pollinator attraction [77]. Therefore, they have a marked effect on outcrossing rates and fitness [17,77,78,79,80,81,82]. The pollen grains released by the two varieties *A.P*. var. *pyrethrum* and *A.P*. var. *depressus* are spheroidal, radially symmetrical, and echinulate with densely arranged spines, which makes them suitable for adherence to insect vectors [83]. The pollinators of the two varieties belonged mainly to Coleoptera and Hymenoptera.

## 3. Materials and Methods

### 3.1. Study Sites

The plant material was collected from three populations for the variety *A.P* var. *depressus*; for the variety *A.P* var. *pyrethrum*, only one population was used for the study due to the rarity of this variety in the study area (Table 5).

The different traits of the biology of reproduction were studied in the field, in the experimental plots in the nursery of the Faculty of Sciences, Dhar El Mahraz, Fez, and in the laboratory of Biotechnology, Environment, Agro-Food and Health of the Faculty of Sciences, Dhar Al Mahraz, Fez.

### 3.2. Population Density

**Figure 4:**
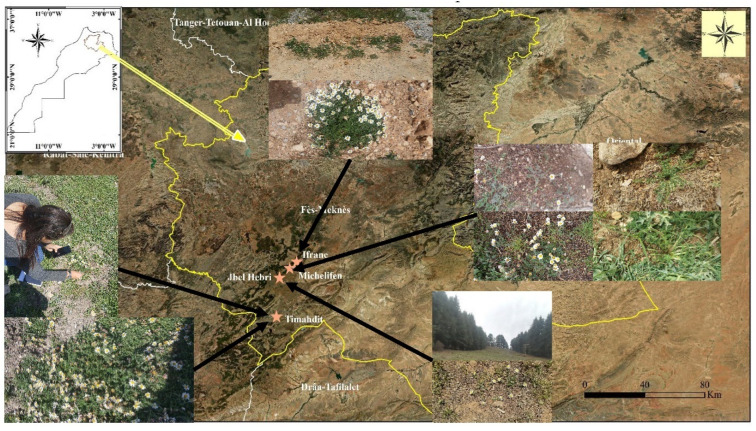
Natural populations of *A.P* var. *depressus* and *A.P* var. *pyrethrum* monitored and sampled.

The density of each population was determined by counting the number of individuals present at the site. The sampling technique adopted was random. Quadrats of 4 m^2^ were randomly placed on a grassy area in the study areas according to the number, topology, and distribution of individuals. A total of 45 quadrats were placed in four stations (Figure 4).

### 3.3. Phenology and Fertility in Natural Conditions

In each population of the two varieties surveyed, flowering and fruiting were monitored between June 2017 and August 2019. During the study period, we quantified the number of flowers or seeds per inflorescence. These two variables allowed us to determine the percentage of flowers that developed seeds in open pollination. For each variety, we then collected flower heads to examine the floral architecture and determine the number of mature and immature seeds developed per head. Photographs were taken to document the floral macromorphology.

### 3.4. Reproduction System

Seeds collected from natural populations of both varieties were sown to initiate an ex situ collection. After seed germination (2–21 days), the seedlings were placed in the field and monitored until flowering. Flower heads emerged between 12 and 14 months after sowing, from which we determined, under experimental conditions, the modalities of reproduction. In particular, we quantified the production of seeds under self-pollination and open pollination.

The sample size was 50 plants. The observations on the reproductive biology were made in 12–14-month-old plantations. At the beginning of the flowering period, the capitula/individuals, in bud, were bagged in a fine mesh netting in order to allow abundant entry of light, humidity, and gas exchange with the outside environment and to prevent visits from pollinators. The bags were regularly shaken during the day to encourage the deposition of pollen on the stigmas. The bags were not removed until the fruit had formed or the flowers were falling. The number of mature and non-mature seeds per capitula was recorded for each treatment:

- A total of 20 capitula bagged at the bud stage to test self-pollination (Lot 1).
- A total of 10 individuals bagged at the bud stage to test intra-individual self-compatibility (Lot 2).
- A total of 20 individuals not bagged to test seed formation in free pollination (Lot 3).

The fructification index (FI) was calculated for each treatment according to the following formula [84]:

(1) $$\mathrm{IF}=\frac{\mathrm{Fr}2}{\mathrm{Fr}1}$$

where Fr1 is the number of flowers and Fr2 is the number of fruits formed.

The difference between the fruiting indices of the different treatments was used to determine the rates of allogamy (TC) and autogamy (TA), which were calculated according to the following formulae [84,85]:

$$TC=\left(\frac{\mathrm{IF}1-\mathrm{IF}2}{\mathrm{IF}1}\right)\times 100\;\;\;\;;\;\;\;\;TA=100-\mathrm{TC}$$

where IF1 is the fructification index of plants in free pollination and IF2 is the fructification index of the bagged plants.

### 3.5. Pollen Morphology

The pollen was mounted in glycerine water and was observed by light microscopy to determine the structure and diameter. Photographs were taken to document the morphology of the pollen.

### 3.6. Pollinators

The capturing of pollinators was favoured during days without strong winds and significant cloud cover. The insect specimens encountered at each site were captured and preserved in ethyl acetate. The taxonomic identification of the insect specimens was carried out by an entomology specialist.

### 3.7. Statistical Analysis

The results were analysed using Graph Prism version 7 and Microsoft Excel 2010 in order to highlight the effect of the various factors and their possible interactions. Statistical processing was performed through an analysis of variance, followed by a Tukey multiple comparison test. A significant difference was considered at *p* < 0.05.

## 4. Conclusions

The results of the experiments described in this work revealed that *Anacyclus pyrethrum* L. is a gynomonoecious, partially self-incompatible species with geitonogamous peripheral flowers and xenogamous central flowers. The pollinators of the two varieties, *A.P* var. *depressus* and *A.P* var. *pyrethrum*, mainly belonged to Coleoptera and Hymenoptera.

## Figures and Tables

**Table 1:** Population density in 2018 and 2019 at different study sites of *A.P* var. *depressus* and *A.P* var. *pyrethrum*.

Stations	Population No	Varieties	Number of Quadrats	Density of Populations/Quadrat (4 m^2^)		Significance
				Year 2018	Year 2019	
Ifrane (Palace Side)	P1	*A.P* var. *depressus*	5	14 ± 1.51	19.4 ± 0.81	**
Michelifn	P2	*A.P* var. *depressus*	10	26 ±1.22	18.8 ± 0.73	***
	P3	*A.P* var. *pyrethrum*	1	5 ± 0	0	***
Jebel Habri	P4	*A.P* var. *depressus*	10	12.8 ± 0.58	8 ± 0.70	***
Timahdite (Tassemagt El Maadane)	P5	*A.P* var. *depressus*	10	10.4 ± 0.74	5.2 ± 0.66	***
	P6	*A.P* var. *pyrethrum*	10	63.8 ± 1.88	55.4 ± 1.43	***

*** Statistically highly significant; ** statistically significant.

**Table 2:** Reproductive performance of natural populations of *A.P*. var. *pyrethrum* and *A.P*. var. *depressus*.

Variables	*Anacyclus pyrethrum* var. *pyrethrum*	*Anacyclus pyrethrum* var. *depressus*	Significance
Number of flowers per capitulum	128.28 ± 27.65	91.2 ± 26.03	**
Number of peripheral flowers/capitula	10.92 ± 1.28	13.15 ± 0.97	**
Number of central flowers/capitula	117.36 ± 27.50	78.05 ± 25.92	**
Number of seeds/capitula	116.98 ± 21.75	81.73 ± 22.45	**
Number of capitula per individual	46.33 ± 10.09	115.49 ± 36.58	***
Number of seeds per individual	5307.46 ± 1501.85	9490.71 ± 4840.36	***
Weight of 100 seeds	0.13 ± 0.01	0.05 ± 0.005	**

*** Statistically highly significant; ** statistically significant.

**Table 3:** Reproduction modalities of the two varieties *A.P*. var. *pyrethrum* and *A.P*. var. *depressus*.

Treatments		Number of Flowers	Number of Seeds	IF (Fruiting Index)	TC (Outcrossing Rate)	TA (Self-Pollination Rate)
Lot 1 (Bagged capitula)	*A.P*. var. *depressus*	64.7 ± 14.90	0	0	100%	0%
	*A.P*. var. *pyrethrum*	65.15 ± 11.81	0	0	100%	0%
Lot 2 (Bagged plants)	*A.P*. var. *depressus*	737 ± 190.10	115.4 ± 22.66	0.16	78.70%	21.29%
	*A.P*. var. *pyrethrum*	458 ± 127.49	81.5 ± 30.51	0.15	79.01%	20.98%
Lot 3 (Free plants)	*A.P*. var. *depressus*	710 ± 166.94	542 ± 150.81	0.75	-	-
	*A.P*. var. *pyrethrum*	439.6 ± 137	353.1 ± 141.86	0.74	-	-

**Table 4:** Orders and families of the various insects recorded on the capitula of *A.P*. var. *pyrethrum* and *A.P*. var. *depressus*.

Population	Order	Suborder	Families	Genus	Species	Photos
*A.P*. var. *pyrethrum*	Hemiptera	Heteroptera	Pentatomidae	*-*	*-*	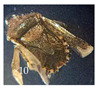
	Hymenoptera	Aculeates	Formicoidae	*Messor*	*Messor* sp.	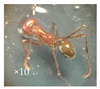
	Coleoptera	Polyphagous	Curculionidae	*Lixus*	*Lixus* sp.	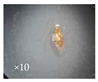
	Coleoptera	Polyphagous	Geotrupidae	*Typhaeus*	*Typhaeus* sp.	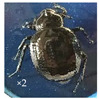
	Hymenoptera	Apocrites	Apidae	*-*	*-*	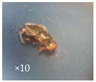
	Coleoptera	Polyphagous	Staphylinidae	*-*	*-*	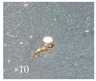
	Hymenoptera	Apocrites	Formicoidae	*Camponotus*	*Camponotus* sp.	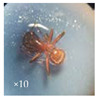
	Coleoptera	Polyphagous	Curculionidae	*Sitophilus*	*Sitophilus* sp.	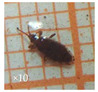
	Lepidoptera	Macrolepidoptera	Sphingidae	*-*	*-*	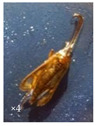
	Coleoptera	Adephagous	Carabidae	*Carabus*	*Carabus* sp.	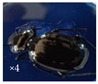
	Araneids	Araneomorphs	Lycosidae	*-*	*-*	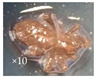
	Lepidoptera	Macrolepidoptera	Sphingidae	*-*	*-*	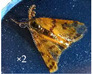
	Lepidoptera	Macrolepidoptera	Sphingidae	*-*	*-*	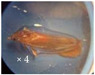
	Lepidoptera	Macrolepidoptera	Sphingidae	*-*	*-*	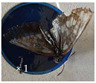
	Hymenoptera	Apocrites	Mutillidae	*-*	*-*	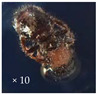
	Orthopteres	Caelifera	Acrididae	*Acridella*	*Acridella* sp.	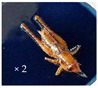
	Orthopteres	Ensiferes	Tettigoniidae	*Tettigonia*	*Tettigonia* sp.	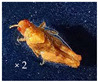
*A.P*. var. *depressus*	Orthopteres	Ensiferes	Tettigoniidae	*Eugaster*	*Eugaster* sp.	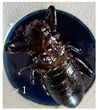
	Hemiptera	Heteroptera	Pentatomidae	*-*	*-*	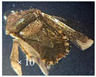
	Coleoptera	Polyphagous	Scarabaeidae	*-*	*-*	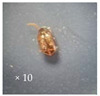
	Hymenoptera	Apocrites	Apidae	*-*	*-*	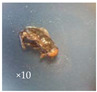
	Coleoptera	Scarabaeoides	Scarabaeidae	*Phyllopertha*	*Phyllopertha horticola*	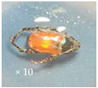
	Dermapteres	Eudermapteres	Forficulidae	*Forficula*	*Forficula* sp.	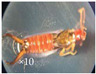
	Orthoptera	Coelifera	Acrididae	*Locusta*	*Locusta* sp.	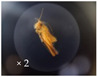
	Coleoptera	Polyphagous	Tenebrionidae	*-*	*-*	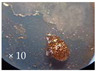
	Coleoptera	Polyphagous	Cerambycidae	*-*	*-*	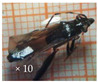

**Table 5:** Coordinates of the harvesting sites.

Harvest Stations	Population No	Latitude	Longitude	Altitude	Varieties
**Ifrane**	P1	33.53178643° N	5.10252178° W	1683 m	*A.P* var. *depressus*
**Michelifn** **(Station Réseau)**	P2	33.41914871° N	5.07954732° W	1955 m	*A.P* var. *depressus*
**Timahdite** **(Tassemagt El Maadane)**	P3 P4	33.14311626° N	5.15923206° W	1948 m	*A.P* var. *depressus* *A.P* var. *pyrethrum*

## Data Availability

Not applicable.

## Abbreviations

IF: fruiting index; TC: outcrossing rate; TA: self-pollination rate; *A.P* var. *depressus*: *Anacyclus pyrethrum* var. *depressus* (Ball.) Maire; *A.P* var. *pyrethrum*: *Anacyclus pyrethrum* var. *pyrethrum* (L.) Link.
